# Bacteriocins as a Promising Antimicrobial Strategy Against Multidrug‐Resistant Pathogens: Mechanisms of Action, Applications in Human and Animal Health, and the Food Industry

**DOI:** 10.1155/bmri/1527677

**Published:** 2026-05-06

**Authors:** Abdo Megra Geda, Aregash Wendimu Tumebo, Solomon Lulie Abey, Gashaw Enbiyale Kasse, Abraham Belete Temesgen, Zerihun Getie Wassie

**Affiliations:** ^1^ Department of The Donkey Sanctuary Project, College of Veterinary Medicine and Agriculture, Addis Ababa University, Bishoftu, Ethiopia, aau.edu.et; ^2^ Department of Veterinary Medicine, School of Veterinary Medicine and Animal Sciences, Wachemo University, Hossana, Ethiopia, wcu.edu.et; ^3^ Department of Veterinary Pathobiology, College of Veterinary Medicine and Animal Sciences, University of Gondar, Gondar, Ethiopia, uog.edu.et; ^4^ Department of Veterinary Clinical Medicine, College of Veterinary Medicine and Animal Sciences, University of Gondar, Gondar, Ethiopia, uog.edu.et; ^5^ Department of Biomedical Science, College of Veterinary Medicine and Animal Science, University of Gondar, Gondar, Ethiopia, uog.edu.et

**Keywords:** antimicrobial agent, application, bacteriocin, mechanisms of action

## Abstract

The global rise of antimicrobial resistance has significantly increased the difficulty treating infectious agents, creating an urgent need for alternative therapeutic strategies. In this context, bacteriocins have emerged as a promising solution, offering a potential alternative to conventional antibacterial therapies. This review explores the potential application of bacteriocins in combating pathogenic bacteria across various sectors, including healthcare and the food industry, while also highlighting mechanisms of action. Bacteriocins are antimicrobial peptides produced ribosomally by certain bacteria and archaea, capable of inhibiting the growth of similar pathogens through distinct modes of action. These peptides have been utilized in both human and veterinary medicine, as well as in agriculture, where they help in preventing unwanted growth of microorganisms. In veterinary practice, bacteriocins are incorporated into products such as udder disinfectants for dairy cattle and topical dermatological formulations for dogs, cats, and horses. Given their broad range of applications, bacteriocins warrant greater consideration by clinicians, veterinarians and industry stakeholders for the prevention and control of microbial infections. Nevertheless, the implementation of robust surveillance systems is crucial for curbing the spread of multidrug‐resistant pathogens, rather than relying solely on conventional antibiotic therapies.

## 1. Introduction

The discovery and advancement of antibiotic therapy in the twentieth century significantly reduced illnesses and mortality in both humans and animals [1]. However, the rise of single‐ and multidrug‐resistant (MDR) pathogens has compromised their effectiveness, leading to the spread of MDR bacteria [2]. This leads to increased treatment costs, morbidity, mortality, hospitalization time, and healthcare expenses [3, 4].

Addressing these challenges requires multifaceted strategies to curb multidrug resistance, particularly by limiting the spread of MDR bacteria. Among the proposed alternatives, Bacteriocins have gained attention as promising substitutes for conventional antibiotics in the fight against drug‐resistant infections [5]. These novel bioactive compounds are being explored to overcome the limitations of declining antibiotic performance, improve treatment outcomes, and provide economically sustainable options in response to the worldwide expansion of antibiotic resistance [6].

Bacteriocins are ribosomally synthesized antimicrobial peptides ranging in size from 2 to over 100 kDa (kilo Dalton). They are produced by a wide range of microorganisms, including gram‐positive and gram‐negative bacteria, as well as certain Achaea [7]. This group constitutes an important category of antimicrobial peptides with wide‐ranging applications in health and agricultural fields [8]. Functioning as biologically active proteins, bacteriocins exert bactericidal activity primarily toward closely related bacterial species and exhibit inhibitory effects against various pathogens under in vitro conditions [8, 9].

To date, approximately 47 bacteriocins have been biosynthesized using *Escherichia coli* (*E. coli*) [10]. In addition, *Bacillus subtilis*, *Lactococcus lactis*, and other lactic acid bacteria (LAB) represent another important group of bacteriocin‐producing bacteria (BPB). These microorganisms are favored for industrial‐scale production because they are generally regarded as safe and facilitate easier downstream purification processes [11, 12].

Among the diverse bacteriocins identified, nisin is one of the most well known and is approved by the Food and Drug Administration (FDA). It has been used as an udder disinfectant in dairy cattle under veterinary care to prevent intra‐mammary infections caused by pathogens such as *Staphylococcus aureus*, *Streptococcus uberis*, and *Streptococcus dysgalactiae* [13]. Furthermore, a nisin‐based formulation, Preva (Kemin Industries, Des Moines, Iowa, United States), containing 25 g/mL active pharmaceutical ingredient (API), has recently been introduced to the American veterinary market. It is indicated for the topical treatment of dermatological conditions associated with bacterial infections in dogs, cats, and horses [14]. Therefore, this review aims to provide updated and essential insights into the potential applications of bacteriocins in inhibiting pathogenic microorganisms, particularly MDR bacteria, compared with conventional antibiotics across various health sectors, highlighting their mechanisms of action and therapeutic effects.

## 2. Bacteriocin

### 2.1. Overview of Bacteriocins

Bacteriocins were first discovered approximately a century ago as cationic antimicrobial peptides. They are generated by various organisms as antimicrobial proteins (AMPs), with a significant portion referred to as antimicrobial peptides due to their small size [15]. These compounds are predominantly synthesized by bacterial ribosomes, with structural genes encoding for AMPs known as bacteriocins [16]. They function by creating pores and disrupting the integrity of target cell membranes, ultimately resulting in cell death [17].

Bacteriocins possess unique characteristics that distinguish them from conventional antibiotics. A key difference lies in their mode of synthesis: bacteriocins are ribosomally produced by bacteria, whereas most antibiotics are synthesized via complex secondary metabolic pathways. Bacteriocins typically exhibit narrow‐spectrum antimicrobial activity, targeting specific or closely related bacterial strains, whereas antibiotics often have broad‐spectrum effects, impacting a wide range of microorganisms, including beneficial microbiota. Due to this myriad of benefits, they are now employed as antibacterial agents [7, 18].

According to this context, LAB are of particular interest due to their antibacterial activity. They are known to produce bacteriocins and have been granted Generally Recognized as Safe (GRAS) status by the American FDA [5]. LABs are well known for producing a variety of bacteriocins with characteristics ideal for pathogen inactivation, including pH and heat stability, nontoxicity, and susceptibility to digestive proteases such as trypsin, chymotrypsin, and pancreatic enzymes. These features allow LAB‐derived bacteriocins to selectively target pathogens while sparing beneficial gut microbiota [8].

Importantly, BPBs are typically resistant to their own antimicrobial peptides. This self‐immunity is achieved through the production of immunity proteins, the use of efflux pumps, or a combination of both mechanisms [19]. The genes responsible for bacteriocin synthesis and immunity are often organized in operon clusters, commonly located on mobilizable genetic elements such as plasmids or chromosomal regions associated with transposons, which facilitates their transfer and molecular study [20].

### 2.2. Classification of Bacteriocins

Multiple classification schemes for bacteriocins have been suggested and revised numerous times, primarily relying on recent structural advancements and an understanding of their modes of action [21]. In this context, and based on the producing organisms, either from gram‐positive bacteria, gram‐negative bacteria, or some archaeal organisms, bacteriocins have been classified into three schemes (Table 1). These classifications of bacteriocins also provide a significant and representative example of each class, offering the advantage of simplicity and applicability for use in various health sectors [16].

**Table 1:** Classification of bacteriocins based on the producers′ organism.

Producer	Example	Source
Gram‐positive bacteria	Nisin, mersadicin, subtilosin, Lacticin 3147,	[22, 23]
	Pediocin RA1, Lactococcin A, Plantaricin ZJ5, sakacin, Carnobacteriocin B12, Helycins, Fermenticin	
	HV6b, Megacin, Zoocin A, Enterocin AS‐48, enterolysin	
Gram‐negative bacteria	Colicins A, B, D, E2–E6, V, and DF13, Cloacin DF13, R‐ and F‐piocins, S‐type piocins, klebicins, azurin, Microcins B17, C7–C51, J25, and E492	[24, 25]
Archaea	Halocin A4, C8, H4, and H6, Sech7a, Sech10, Sulfolobicins	[26, 27]

#### 2.2.1. Bacteriocins From Gram‐Positive Bacteria

Bacteriocins produced by gram‐positive bacteria exhibit a broad spectrum of diversity, encompassing diverse sizes, physicochemical properties, structures, and inhibitory spectra [28]. Due to this extensive diversity, numerous classification schemes for this group of bacteriocins have been proposed in several studies [16, 29]. They are categorized into three classes: Class I, II, and III, based on their structural and genetic properties [8, 30].

Class I bacteriocins are small peptides (< 5 kDa) composed of 19 to 50 amino acids. They belong to a diverse group of ribosomally synthesized and posttranslationally modified peptides (RiPPs), which include lantibiotics, lasso peptides, linear azole‐containing peptides (LAPs), thiopeptides, sactipeptides, and glycocins. These peptides are generally heat‐stable and undergo extensive posttranslational modifications, leading to the formation of unusual amino acids such as lanthionine and methyllanthionine [16, 31].

Functionally, Class I bacteriocins act by inhibiting essential catalytic enzymes required for bacterial survival [15]. This class is further subdivided into three subclasses: Class Ia, Ib, and Ic. Class Ia bacteriocins include nisin, subtilin, epidermin, gallidermin, and mersacidin. These are typically flexible, elongated peptides that are positively charged and have hydrophobic properties, facilitating pore formation in bacterial membranes [15].

In contrast, Class Ib bacteriocins, such as Lactacin 481, actagardine, cytolysin, and cinnamycin, are generally globular and rigid in structure, often carrying a negative charge. Class Ic bacteriocins, known as sactipeptides, are characterized by unique sulfur‐to‐carbon linkages. Representative examples include subtilocins A (also known as subtilomycins) from *B. subtilis*, thuricin S from *Bacillus thuringiensis*, and estercticin A from the psychrophilic *Clostridium estertheticum* [8].

Class II bacteriocins are small (< 10 kDa), flexible peptides that typically exhibit an amphiphilic *α*‐helical structure [17]. Unlike Class I bacteriocins, they do not undergo extensive posttranslational modifications and therefore lack unusual amino acid residues. These peptides are generally stable across a wide range of pH values and temperatures. Their structural properties enable them to traverse the bacterial cell wall and exert antimicrobial activity by increasing membrane permeability, ultimately causing leakage of intracellular components and cell death [29].

Class II bacteriocins display potent activity against *Listeria monocytogenes* and are subdivided into four subclasses: Subclass IIa, IIb, IIc, and IId, based on structural characteristics and functional properties [32]. Subclass IIa bacteriocins, often referred to as Pediocin PA‐1, Sakacin A, Enterocin A, Leucocin A, BavaricinMN, and DivercinV41. These are small (37–48 amino acids), heat‐stable, unmodified peptides with a net positive charge. They are highly conserved and hydrophilic, featuring a charged N‐terminal region responsible for binding to the targeted cell membrane. They also have a more variable hydrophobic or amphiphilic C‐terminal region that forms a hairpin‐like structure, which penetrates the target cell membrane, leading to cell death [33].

Subclass IIb bacteriocins, such as lactococcin G, lactococcin M, Plantaricin NC8, EF and JK, are among the best‐characterized two‐peptides, both functionally and structurally [30]. These bacteriocins act synergistically and are primarily active against gram‐positive bacteria. They are frequently produced by LABs, highlighting their importance in food preservation and biocontrol applications [34].

Subclass IIc bacteriocins include cyclic peptides such as enterocin AS‐48, acidocin B, and gassericin A. This group is less clearly defined and may also encompass certain larger antibacterial proteins that were previously classified as Class III bacteriocins. These molecules differ structurally from the smaller peptides of Subclass IIa and may exhibit different mechanisms of action. Aureocin A53, Lactococcin A, and Bactofencin A are also another typical group of Subclass IId bacteriocins. As others, Subclass IId bacteriocins include single‐peptides, nonpediocin‐like bacteriocins such as aureocin A53, lactococcin A, and bactofencin A, which possess unique structural and functional features within Class II [30, 35].

Class III bacteriocins are high–molecular‐weight proteins (> 30 kDa) that are heat‐labile and generally lack post translational modifications. Unlike the smaller bacteriocins of Classes I and II, these proteins exert their antimicrobial activity primarily by targeting and disrupting the bacterial cell wall, ultimately leading to cell death. Class III bacteriocins are further divided into two subclasses: Subclass IIIa and IIIb, based on their mode of action. Subclass IIIa bacteriocins are bacteriolytic, meaning they cause cell lysis. Representative examples include Enterolysin A from *Enterococcus faecalis* and megacins A‐192 from *Bacillus megaterium*. In contrast, subclass IIIb bacteriocins are nonlytic and inhibit bacterial growth without causing direct cell lysis. Examples include megacin A‐216 and Helveticin J from *Lactobacillus helveticus* [34, 36].

#### 2.2.2. Bacteriocins From Gram‐Negative Bacteria

Gram‐negative bacteriocins are primarily classified into two major groups based on their molecular size [37]. The first group comprises microcins, which are small peptides with a molecular mass of less than 10 kDa and are mainly produced by members of the Enterobacteriaceae family, such as *E. coli* [38, 39]. Microcins are generally highly stable and can withstand elevated temperatures, extreme pH conditions, and the action of protolytic enzymes [40–42].

The second group includes colicin and colicin‐like bacteriocins, which are also produced by gram‐negative bacteria. Colicins are among the most extensively studied bacteriocins and typically range from 30 to 80 kDa in size [40, 41]. In addition to colicins, high–molecular‐weight bacteriocins known as tailocins are recognized within this category. Tailocins are multisubunit protein complexes that structurally resemble the contractile tails of bacteriophages and function as potent antibacterial agents [26, 39].

#### 2.2.3. Bacteriocins of Archaeal Organisms

As with the bacteriocins of gram‐positive and gram‐negative bacteria, some archaeal species produce bacteriocins known as archaeocins or halocins [43]. These groups of bacteriocins exhibit diversity in structure, range of activity, and ecological functions, as compared with that of bacteriocins produced by bacteria [44].

Halocins are among the best bacteriocin‐resembling antimicrobial peptides produced by halophilic archaea [45]. They are effective against other haloarchaea, including species of the genera *Haloferax*, *Halobacterium*, and *Natrinema*, as well as some crenarchaea, such as Sulfolobus [16]. However, there are no current reports demonstrating the antimicrobial activity of halocins against bacterial infections, underscoring their narrow spectrum of activity as bacteriocins produced by bacteria [46].

Halocins can be categorized into two main types: low‐molecular weight hydrophobic peptides (< 10 kDa), which are also called microhalocins, and high‐molecular weight halocins (> 10 kDa). Microhalocins are relatively insensitive to heat and organic solvents, whereas high‐molecular weight proteins are susceptible to heat, proteases, and desalination. Examples of microhalocins include HaIS8, HaIR1, HaIC8, HaIU1, HaIH6, Sech7a, and Sech10. Similarly, Halocin H4 and Halocin H6 are examples of high‐molecular mass halocins [47, 48].

### 2.3. Bacteriocins′ Mechanisms of Action

Bacteriocins are highly potent bactericidal agents that act rapidly to eliminate pathogenic bacteria. They exert their antimicrobial effects by forming pores in the bacterial cell envelope, which leads to the leakage of essential intracellular components and ultimately inhibits bacterial growth [6, 49]. Depending on their primary structure and target specificity, bacteriocins employ diverse mechanisms of action, some resembling those of conventional antibiotics. Although certain bacteriocins primarily target the cytoplasmic membrane, others penetrate into the cytoplasm and interfere with gene expression, DNA replication, or protein synthesis [16].

Colicins are among the most extensively studied bacteriocins, which provide a well‐characterized model of bacteriocin‐mediated killing in closely related bacteria [40]. Colicins are encoded by colicin gene clusters. These clusters typically comprise three components: (a) a structural gene encoding the toxin, (b) an immunity gene encoding a specific immunity protein that protects the producer cell by binding to and neutralizing the toxin, and (c) a lysis gene that facilitates toxin release through cell lysis. Target recognition is mediated by a receptor‐binding domain within the colicin protein that attaches to specific receptors on susceptible cells. Once internalized, colicins kill target bacteria through mechanisms such as DNase, rRNase, or tRNase activity, or by forming pores in the cell membrane [41].

Microcins represent another important group of bacteriocins with significant antibacterial activity. They are encoded by conserved genetic systems and are initially synthesized as precursor peptides containing a core peptide and an N‐terminal leader sequence. After cleavage of the leader peptides, mature microcins are exported via ATP‐binding cassette (ABC) transporter‐like systems or dedicated efflux pumps. Upon reaching target cells, microcins either interact with the inner membrane or bind to essential intracellular enzymes, ultimately leading to the bacteria cell death [38, 42].

Halocins are a group of bacteriocins that include two main types: microhalocins and high–molecular‐weight halocins, each with distinct mechanisms of action for inhibiting target organisms. Microhalocins typically act by disrupting the integrity of the cell membrane. Due to their hydrophobic nature, they can readily insert into the lipid bilayer of the target cell. Once embedded, they form pores that compromise membrane stability. This pore formation leads to the leakage of essential ions and molecules, ultimately resulting in cell lysis and death [47].

In contrast, high‐molecular‐weight halocins tend to act through more specific intracellular targets. Rather than disrupting the membrane directly, they interfere with critical cellular processes like cell wall synthesis, DNA replication, or protein synthesis. Because of this targeted mode of action, high–molecular‐weight halocins are more specific and can be particularly effective against certain strains or species of archaea [16, 48].

Bacteriocins exert their antimicrobial effects primarily through initial interactions with the cell membrane. This interaction typically occurs either by binding to specific membrane‐bound receptors or through electrostatic attraction between positively charged amino acid residues of bacteriocins and the negatively charged phospholipid on the bacterial cell surface [17]. Once attached, bacteriocins may alter enzyme activity, inhibit spore germination, inactivate anionic transport systems, or create selective or nonselective pores in the cytoplasmic membrane. Overall, bacteriocins function as potent antimicrobial agents through multiple mechanisms, including inhibition of cell wall synthesis, disruption of membrane integrity, interference with DNA replication, and suppression of protein synthesis [49].

#### 2.3.1. Action on Cell Wall Synthesis and Disruption of Bacterial Cell Membrane

Some Class I bacteriocins, like nisin, have demonstrated remarkable effectiveness against their target strains, exhibiting antimicrobial activity even at nanomolar (nM) concentrations [50]. Nisin attaches to the Lipid II molecule, the primary carrier of peptidoglycan subunits from the cytoplasm to the cell wall, thereby hindering bacterial cell wall formation through enzyme activity [51]. On the other hand, nisin has a direct effect on the targeted bacterial cell membrane, in which it can penetrate the cell membrane and induce pore formation. This mechanism of nisin leads to the release of essential ions such as potassium (K^+^) and magnesium (Mg^2+^), and also amino acids, which are required for bacterial survival, ultimately resulting in cell death [52].

**Figure 1:**
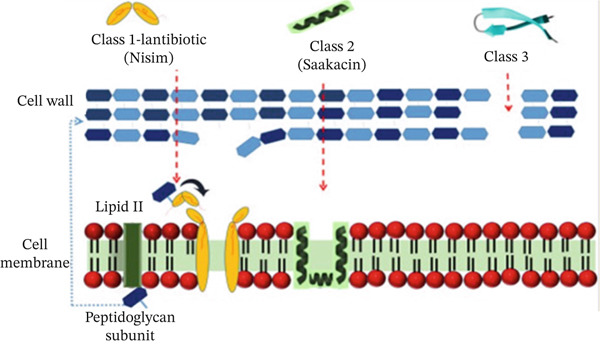
Mechanism of bacteriocins in preventing cell wall synthesis and cell membrane disruption. Nisin binds to Lipid II molecules, hindering the synthesis of peptidoglycan and then creates pores in bacterial cell membranes [52]. Similarly, Class II bacteriocin (sakacin) penetrates the cell wall and binds to the pore‐forming receptor in the mannose–phosphotransferase system, inducing depolarization of the cell membrane and ultimately resulting in cell death [30]. Class III bacteriocins, such as zoocin A, directly target the cell wall by cleaving the peptidoglycan cross‐links [36, 48].

Additionally, because they possess an amphiphilic helical structure, Class II bacteriocins penetrate the membrane of the target organism and disrupt cell contents by interfering with the proton motive force, among other activities [30]. Similarly, Class III bacteriocins are subdivided into two subclasses: IIIa (lysostaphin and entrolysin A), or bacteriolysins; and IIIb. Subclass IIIa, known as bacteriolysins, can directly catalyze the cell wall hydrolysis, leading to bacterial cell death [36]. Another group of Class III bacteriocins (IIIb), nonbacteriolytic ones, works by preventing the cells from absorbing glucose, starving them and altering the membrane potential. Thus, they inhibit the target bacteria′s ability to create protein and DNA, which are essential for the survival of bacterial pathogens (Figure 1) [48].

#### 2.3.2. Inhibition of Bacterial Genetic Synthesis

Several bacteriocins targeting gram‐negative bacteria disrupt cellular metabolism by interfering with DNA replication, RNA transcription, and protein synthesis [15]. To exert these effects, they must first traverse both the outer and inner membranes of the target cell through specific transporting mechanisms. Once inside the cytoplasm, they degrade essential intracellular components, including DNA and RNA, often through proteolytic enzyme activity. Notably, this mode of action is generally regarded as nontoxic to eukaryotic cells, highlighting their potential as selective antimicrobial agents [53].

Among bacteriocins, microcin such as microcin J25 (MccJ25) and microcin B17 (MccB17) are well characterized for their ability to interfere with transcription and DNA replication, respectively. MccJ25 inhibits RNA polymerase, whereas MccB17 targets DNA gyrase. The antibacterial activity of both MccJ25 and MccB17 relies on a highly coordinated, receptor‐mediated translocation mechanism that enables them to cross the outer and inner membranes of gram‐negative bacteria. MccJ25 initially binds to the ferrichrome receptor FhuA on the outer membrane, hijacking this nutrient uptake system to gain entry into the cytoplasm. In contrast, MccB17 utilizes the OmpF porin, a general diffusion channel, to pass through the outer membrane [30].

After reaching the periplasm, both microcins must traverse the inner membrane to access their intracellular targets. MccJ25 requires the inner membrane protein SbmA, an ABC transporter‐like protein, for translocation into the cytoplasm. Similarly, MccB17 depends on inner membrane transport systems, including SbmA, to facilitate its import. Once inside the cytoplasm, MccJ25 binds within the secondary channel of RNA polymerase, blocking nucleotide entry and inhibiting transcription. Meanwhile, MccB17 stabilizes the cleavable complex formed by DNA gyrase and DNA, leading to double‐stranded DNA breaks and inhibition of DNA replication, ultimately resulting in bacterial cell death [16, 54].

**Figure 2:**
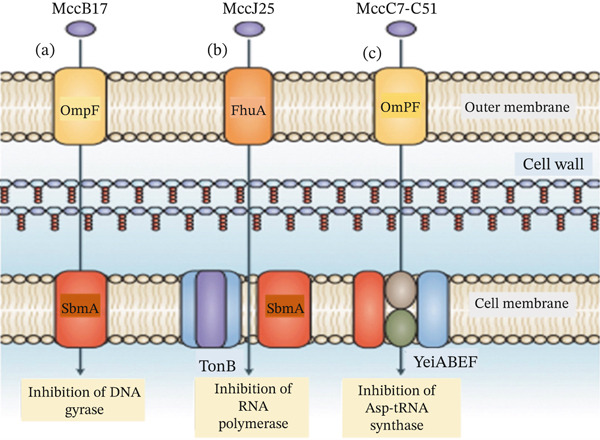
Inhibitory effects of bacteriocins on bacterial DNA, RNA, and protein synthesis. (a) MccB17 inhibits DNA gyrase, thereby disrupting DNA replication. (b) MccJ25 targets RNA polymerase, blocking transcription and consequently suppressing bacterial gene expression. (c) MccC7–C51 inhibits protein synthesis and interferes with DNA replication, ultimately leading to bacterial cell death [16, 54].

Another example is microcin C7–C51 (MccC7–C51), which exerts toxicity by inhibiting protein synthesis and interfering with DNA replication. Its activity can also be toxic to the producing cell, and these combined effects contribute to the cell death (Figure 2) [54]. Similarly, bacteriocins such as cloacin DF13 and colicins E3–E6 exhibit ribonuclease activity directed against 16S rRNA. Group E colicins possessing endonuclease activity further disrupt translation by cleaving the 3 ^′^ ends of the coding region of 16S rRNA. In addition, certain colicins, including D and E5, function as tRNases, causing depletion of specific tRNAs. This depletion impairs protein synthesis and ultimately leads to the death of susceptible bacterial strains [55].

## 3. Application of Bacteriocins

Extensive research on the antibacterial properties of bacteriocins has enabled their development for various practical applications. Broadly, these applications can be categorized into two major areas: medical and veterinary uses and applications in the food industry [56]. In medicine and animal health, bacteriocins are explored as alternative complementary antimicrobial agents. In the food sector, they are widely utilized in food production and preservation to inhibit the growth of undesired microorganisms, thereby enhancing food safety and extending shelf life [56, 57].

### 3.1. Bacteriocins in Human Health

#### 3.1.1. As Antimicrobial Agents

Bacteriocins are an important class of antimicrobial peptides with broad potential in medical applications, particularly for combating antibiotic‐resistant bacteria [58]. Their growing appeal as alternatives to conventional antibiotics is largely attributed to their favorable biological characteristics. These include a strong safety profile, rapid clearance from the body, and stability during passage through the gastrointestinal transit. In addition, bacteriocins are environmentally friendly and exhibit selective antimicrobial activity, targeting pathogenic bacteria while causing minimal disruption to beneficial gut microbiota. This specificity supports improved public health outcomes and enhances their suitability for practical human use [31, 57]. Collectively, these properties make bacteriocins promising therapeutic agents for combating clinically significant pathogens and limiting the development of antibiotic resistance, distinguishing them from conventional antibiotics (Table 2) [59].

**Table 2:** A comparison between bacteriocins and antimicrobials.

Criteria	Bacteriocins	Antibiotics
Biosynthesis	Primary metabolite	Secondary metabolite
Size	15–80 kDa	0.3–0.5 kDa
Active spectrum	Narrow to high	Narrow to broad
Active pH Range	Wide range	Small range
Thermal stability	High	Low
Mode of action	Primarily targets the cell membrane, resulting in pore formation	Often targets the cell wall, as well as protein or nucleic acid synthesis
Color, taste, and odor	No	Yes
Adverse effect	No	Yes
Application	In the food and clinical sector	In the clinical sector
Causing resistance	Low	High
Toxicity	Low	Low to High

*Note:* Source: [57, 59].

#### 3.1.2. In Cancer Prevention

Numerous bacteriocins have been described in the prevention of cancer by selectively targeting cancer cells [59]. For instance, microcin E492 and colicins (A, D, E1, E2, and E3), produced from gram‐negative bacteria, are prime examples of bacteriocins that have displayed cytotoxic effects against malignant human cell lines [60]. Another instance is the bacteriocin azurin, produced by a strain of *Pseudomonas aeruginosa*, which has been explored as a potential anticancer agent due to its specific binding to human cancer cells and subsequent cytotoxic and apoptotic effects without apparent impact on normal cells. They combat cancer cells by inducing apoptosis and/or depolarizing the cell membrane, thereby altering cell permeability [59]. Similarly, nisin, produced by gram‐positive bacteria, has been shown to promote DNA fragmentation and apoptosis. It also reduces cell proliferation by inducing cell cycle arrest in head and neck squamous cell carcinoma (HNSCC) cells [61].

#### 3.1.3. In Birth Control and Women′s Care

Different bacteriocins possess potential as spermicidal agents due to their ability to impact sperm motility [37]. Among these, nisin, subtilosin, and lacticin 3147 stand out as bacteriocins that have demonstrated spermicidal properties by reducing or altering human spermatozoa motility, underscoring their potential as contraceptives for birth control [37, 62].

Another notable bacteriocin is fermenticin HV6b, a Class IIa antimicrobial peptide generated by *Lactobacillus fermentum* HV6b MTCC 10770 isolated from the human vaginal ecosystem. This bacteriocin serves a dual purpose: It inhibits the growth of bacteria such as *Gardnerella vaginalis*, *Mobiluncus*, Staphylococci, and Streptococci, which cause human vaginal infections, while also immobilizing sperm and exhibiting spermicidal activity. To develop vaginal creams that can both prevent microbial infections and act as a form of contraception, a novel formulation containing fermenticin HV6b or *L. fermentum* HV6b can be utilized either alone or in combination [63].

#### 3.1.4. Use of Bacteriocins in Immunomodulators

The full understanding of bacteriocins′ impact on immunomodulation remains elusive. However, it is firmly established that the concentration of bacteriocin employed influences the modulation of the immune system [64]. These mechanisms of immune response activation by bacteriocins complement their bactericidal effect, thereby bolstering host defense, particularly during infections. Among bacteriocins, nisin stands out as one of the oldest and most extensively utilized bacteriocins in immunoregulatory effects [4].

Studies have shown that short‐term administration of nisin as a dietary supplement increases the levels of CD4+ and CD8+ T lymphocytes (LTs) while reducing B lymphocytes (LBs) in the bloodstream [65]. Prolonged usage leads to a restoration in LT levels to normal, a sustained decrease in LB, and an augmentation in the number of macrophages and monocytes. In vitro experiments demonstrate that high concentrations of nisin administered to porcine peripheral blood mononuclear cells (PBMCs) enhance the proliferation of both CD4+ and CD8+ cells as well as the production of cytokines IL‐1*β* and IL‐6 [66].

Moreover, elevated concentrations of nisin in neutrophils trigger the activation of extracellular traps and elevate intracellular superoxide levels [65]. Interestingly, although nisin exhibits minimal antibacterial activity against gram‐positive bacteria in vitro, its administration to animals with both gram‐positive and gram‐negative infections significantly reduces the host′s bacterial load. Tests using human PBMCs reveal that chemokines (MCP‐1, IL‐8, and Gro‐*α*) responsible for inhibiting the production of proinflammatory TNF‐*α* contribute to nisin′s protective effects. In this regard, nisin demonstrates superior potency compared with the human antibacterial peptide LL‐37 [67].

The immunomodulatory effects of nisin extend even to nonimmune cells. Nisin enhances intracellular lysozyme levels in bovine mammary epithelial cells and promotes its release into the extracellular environment [68]. Bacteria acting as immune‐modulating agents have shown anti‐inflammatory capabilities in injured or infected tissue. Nisin inhibits TNF‐*α* production in LPS‐stimulated PBMCs, thereby reducing the inflammatory response [67].

In porcine PBMCs infected with *E. coli*, nisin mitigates the inflammatory response, particularly the generation of IL‐6 [66]. By promoting the negative regulation of TNF‐*α* production, which aids in the healing of intramammary tissue, this anti‐inflammatory action is replicated in bovine mammary gland epithelial cells. Given that endometritis is a common postpartum health issue within the first 3 weeks after delivery, nisin administration protects cattle from endometritis induced by experimental *S. aureus* infection. In this context, bacteriocins facilitate a reduction in proinflammatory cytokines and an increase in anti‐inflammatory cytokines [69].

Studies have revealed that bacteriocins produced by *Lactobacillus rhamnosus* strain L34 and ATCC 53103 reduce postoperative side effects, such as inflammation in tissue injuries like fractures. They also aid in managing experimental *S. aureus* intra‐articular infection [70]. Proinflammatory cytokines TNF‐*α*, IL‐6, and C‐reactive protein were diminished in rabbit models of knee replacement surgery and mandible fracture repair by bacteriocins from *L. rhamnosus*. Additionally, these bacteriocins impede biofilm formation and promote tissue healing to prevent experimental *S. aureus* infection. These findings suggest that bacteriocins could be effective tools in reducing orthopedic postoperative infections [71].

Recent research indicates that the bacteriocin coagulin, expressed by the LAB *Pediococcus pentosaceus* strain SL001, enhances grass carp immunity and accelerates development while aiding in the eradication of infections in tissue lesions such as fractures. Bacteriocins produced by *P. pentosaceus* upregulate immunoglobulin M (IgM) and Complement 3 (C3) while downregulating interleukin‐8 (IL‐8). Furthermore, *P. pentosaceus* promotes grass carp development and assists in infection eradication [72].

### 3.2. Bacteriocins in Animal Health Sector

Bacteriocins are used in various veterinary applications to prevent and control bacterial infections responsible for significant animal diseases [72]. In addition to their antimicrobial role, they also act as growth promoters in livestock by modulating the gastrointestinal microbiota, supporting the growth of beneficial bacteria while inhibiting harmful ones, thereby improving growth rates and reducing mortality [59, 73].

To highlight further, specific bacteriocins, such as nisin, have been effectively employed to combat respiratory tract infections caused by *S. aureus* in animal models and *Streptococcus species* [63]. Injectable formulations of bacteriocins such as lacticin 3147 have been investigated for controlling mastitis‐causing bacteria, with some studies reporting up to 99.9% effectiveness against these pathogens. For instance, products like Mast Out developed by ImmuCell Corporation (Portland, Maine, United States) serve as intramammary alternatives to traditional antibiotics for treating mastitis in lactating dairy cows [74]. Similarly, teat seal from Cross Vetpharm Group Ltd. (Dublin, Ireland) finds widespread use in cow farms [75].

Nisin has also shown synergistic effects with Cefazolin against mastitis‐causing pathogens such as *S. aureus* and *E. faecalis*. This combination has been demonstrated to be effective against these pathogens, thereby enabling a reduction in antibiotic dosage [75]. AP‐CECT7121, derived from the *E. faecalis* strain CECT7121, represents another potential antimastitis peptide. It has exhibited efficacy in vitro against *S. aureus*, *S. dysgalactiae*, *S. uberis*, and *Streptococcus agalactiae* strains isolated from dairy cattle with mastitis [76].

Moreover, purified or semipurified bacteriocins hold promise as antimicrobial agents when added directly to animal feed as anti‐infective supplements. This approach could effectively protect animals such as swine and poultry production from both animal and food‐borne pathogens. This strategy not only reduces the emergence of antibiotic‐resistant bacteria but also mitigates associated financial losses and adverse impacts on human health [21].

Divercin AS7 has demonstrated efficacy in controlling pathogenic bacterial strains such as *Campylobacter species*, *Salmonella enterica* Typhimurium, and *Clostridium perfringens* in chicken or swine [77]. Additionally, another bacteriocin, LFB 112 produced from *B. subtilis*, has exhibited in vitro activity against the growth of *E. coli*, *S. aureus*, *Salmonella enteritidis*, *Pasteurella multocida*, and *Streptococcus equi* subsp. *zooepidemicus* [32]. Enterocin E‐760 isolated from chicken ceca has inhibited the growth of several gram‐positive and gram‐negative bacterial pathogens in animals [21]. Overall, as extensive in vitro and in vivo studies have evaluated the therapeutic potential effect of bacteriocins, they are more widely utilized in livestock, poultry, and small animal sectors for the prevention of various infectious agents (Table 3) [72].

**Table 3:** Veterinary applications and some potential uses of bacteriocins.

Bacteriocin	Producer	Potential use	Source
Nisin	*Lactococcus lactis* spp. Lactis	Used as a food preservative to inhibit food‐borne pathogens. It also treats infectious caused by *S. pneumoniae*, bovine mastitis, and peptic ulcer disease.	[8, 63, 75]
Lacticin 3147	*L. lactis* spp. Lactis	Treats mastitis in cattle.	[75]
Enterocin AS‐48	*Enterococcus faecalis spp*.	Used as a biopreservative in food product to inhibit uropathogenic enterococci.	[78]
Enterocin CRL35	*Enterococcus faecium* CRL35	Used to treat infections caused by both gram‐positive and gram‐negative pathogens when hybridized with microcin V.	[79]
Staphylococcin 188	*Staphylococcus aureus* AB188	Exhibits both anti‐enterococcal and antiviral properties.	[9]
Mutacin B‐Ny266	*Staphylococcus mutans*	Demonstrated effectiveness against bacterial infections caused by methicillin‐resistant staphylococcus species.	[80]
Microcins J25 and J24	*Escherichia coli*	Treats *E. coli* and Salmonella infection in poultry.	[81]
Colicins E1, E4, E7, E8, K, and S4	*E. coli*	Treats hemorrhagic colitis and hemolytic uremic syndrome caused by *Escherichia coli* O157:H7.	[82]

### 3.3. Bacteriocins in the Food Industry

Bacteriocins offer an appealing alternative to chemical preservatives as natural antibacterial agents, addressing the increasing consumer demand for safe, minimally processed, ready‐to‐eat foods [58]. They lack taste, smell, or color, making them suitable for incorporation into food products without altering their sensory qualities [5].

Many bacteriocins exhibit resistance to high temperatures, low pH levels, and various salt concentrations. Due to these biological characteristics, bacteriocins are increasingly utilized as food preservatives [57]. These benefits include extending the shelf life of foods and food products by inhibiting the growth of spoilage microorganisms [8, 83]. For example, bacteriocins such as plantaricin ZJ5, produced by *Lactobacillus plantarum*, and sakacin P and sakacin X, produced by *Lactobacillus curvatus*, have shown significant inhibitory activity against *L. monocytogenes* [7]. They also provide additional protection during temperature abuse scenarios, reducing the risk of foodborne pathogen transmission along the food chain. This mitigates financial losses due to food spoilage, recalls, or outbreaks. Furthermore, the use of bacteriocins enables the adoption of less aggressive food processing methods without compromising food safety, thereby preserving nutrients, vitamins, and other essential components [7].

According to Alvarez‐Sieiro [8], nisin, for example, holds the distinction of being the only bacteriocin that has been granted a biopreservative license. It is utilized in various commercial formulations, such as ChrisinTM (Chris Hansen), NisaplinTM (Danisco), and DelvoNis (DSM), commonly employed in the dairy industry to prevent clostridia and postprocessing contamination from *Listeria* strains [84].

Similarly, nisin and other well‐known bacteriocins have demonstrated efficacy in inhibiting numerous pathogens or spoilage bacteria across diverse food matrices, including dairy products, meat, fish and seafood, juices and beverages, fruits, vegetables, and cereals [57, 85]. They can also facilitate the early ripening of cheddar cheese by inducing premature cell lysis in the starter cultures and releasing intracellular enzymes into the cheese matrix [86].

Furthermore, bacteriocins can be incorporated into food products as semi‐purified chemicals or bioactive powders, often containing a blend of antimicrobial substances like bacteriocins, organic acids, and so on. [6, 74]. These powders are typically produced by cultivating the producer strain in a suitable growth medium, followed by heat treatment to inactivate the bacteria and subsequent drying of the material [57]. Commercial bioactive powders like MicroGard fermentates (Dupont, Wilmington, Delaware, United States) and DuraFresh products (Kerry, Tralee, Ireland) are available on the market. These products exhibit efficacy against yeasts, molds, and both gram‐positive and gram‐negative bacteria. Additionally, bacteriocins can be incorporated into food packaging films to prevent food spoilage or microbial growth during storage [87].

## 4. Advantages and Disadvantages of Bacteriocins

Bacteriocins possess numerous characteristics that make them attractive candidates as novel antimicrobial agents for human and veterinary medicine, as well as for applications in the food industry [15, 56]. They are active against clinically significant pathogens, including MDR bacteria, and often display stability across a broad range of temperatures. In addition, bacteriocins generally exhibit low cytotoxicity toward eukaryotic cells in vitro. For instance, nisin has demonstrated high LD_50_ values in animal studies (> 1000 mg/kg orally in rodents), supporting its safety profile. Their genetic determinants also allow bioengineering through targeted manipulation of bacteriocin genes, offering opportunities to enhance activity, stability, or spectrum [88].

A notable advantage of bacteriocins is their single‐strike kinetics, whereby a single molecule can be sufficient to kill a target cell, either by forming pores in the membrane or by disrupting essential enzymatic functions such as DNase or RNase activity. In contrast, conventional antibiotics typically require sustained concentrations to inhibit or eliminate pathogens by interfering with cellular processes. Furthermore, Bacteriocins are usually degraded rapidly in the body and, owing to their highly specific mechanisms of action, are considered less likely to promote widespread resistance [18].

Despite these advantages, bacteriocins also present several limitations. Many exhibits a narrow antimicrobial spectrum, higher doses may be required to effectively combat MDR pathogens. Production costs can be high, and purification processes often result in low recovery yields, compromising product quality [89]. Their susceptibility to proteolytic enzymes significantly restricts oral administration unless protective delivery systems (e.g., encapsulation, liposomes, and nanoparticles) are employed. Resistance to bacteriocina may also arise, either through acquired mechanisms in previously susceptible strains or through intrinsic resistance present in certain food spoilage or pathogenic bacterial species. Moreover, compared with many chemically stable antibiotics, bacteriocins face notable pharmacokinetic challenges [90]. Research on their safety, pharmacokinetics, pharmacodynamics, and immunogenicity remains limited [91].

## 5. Conclusions and Recommendations

The emergence of bacteriocin represents a modern and promising strategy as an alternative to conventional antibiotics. Owing to their remarkable stability, structural diversity, specific targeting ability, low cellular toxicity, and minimal side effects, bacteriocins demonstrate considerable potential as effective antimicrobial agents. Their selective mode of action allows them to target pathogenic bacteria while sparing beneficial microbiota, further supporting their therapeutic value. When used in combination with conventional antibiotics, bacteriocins often exhibit a synergistic effect. This synergy can enhance antibiotic efficacy, reduce the required dosage of more toxic antibiotics, and potentially limit associated side effects. Moreover, such combinational approaches may help delay or prevent the emergence of antibiotic‐resistant strains. Unlike many forms of antibiotic resistance, resistance to bacteriocins is not typically genetically predetermined, which further strengthens their appeal as alternative therapeutic agents. In human health, bacteriocins may also contribute to the activation of immune responses and enhancement of host defenses, particularly during infections. In veterinary medicine, both topical and injectable formulations of bacteriocins could be incorporated into integrated strategies for microbial control and disease prevention. Beyond clinical settings, bacteriocins hold significant promise in the food industry, where they can inhibit the growth of undesired microorganisms, particularly in food production and preservation. However, further research is required to fully elucidate their role in innate and adaptive immunity and explore potential structural or functional modifications that could improve their efficacy in controlling infections and diseases.

## Author Contributions

Conceptualization, writing—original draft, writing—review and editing, and visualization, A.M.G.; validation, data curation, and visualization, A.W.T.; general supervising, validation, and visualization, S.L.A.; visualization and validation, G.E.K., A.B.T., and Z.G.W.

## Funding

No funding was received for this manuscript.

## Disclosure

All authors have read and agreed to the published version of the manuscript.

## Ethics Statement

The authors have nothing to report.

## Consent

The authors have nothing to report.

## Conflicts of Interest

The authors declare no conflicts of interest.

## Data Availability

The data used to support this review have been included within the article.
